# Supplementary material for: Germline Whole-Gene Deletion of FH Diagnosed from Tumor Profiling
Source: Int J Mol Sci. 2021 Jul 26;22(15):7962. doi: 10.3390/ijms22157962 (PMC8347438; doi:10.3390/ijms22157962)
Supplement: Supplementary file 1 [file ijms-22-07962-s001.zip › ijms-1291537-SI.pdf]

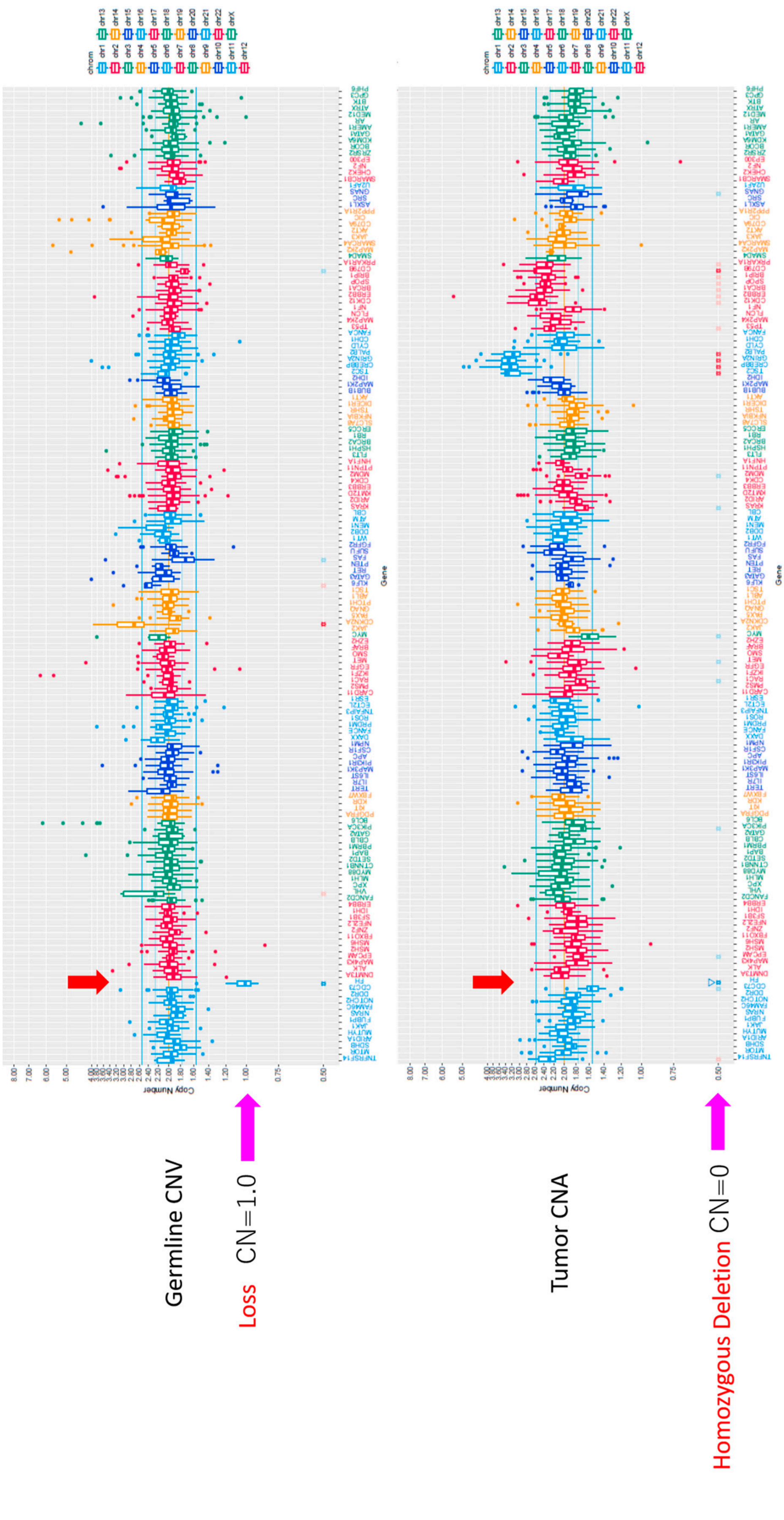

Figure S1. Results of germline and tumor profiling via panel sequencing. The FH gene loss (CN = 1.0) was confirmed in germline, and the homozygous deletion (CN = 0) was confirmed in the tumor. CN, copy number; CNV, copy number variant; CNA, copy number alteration

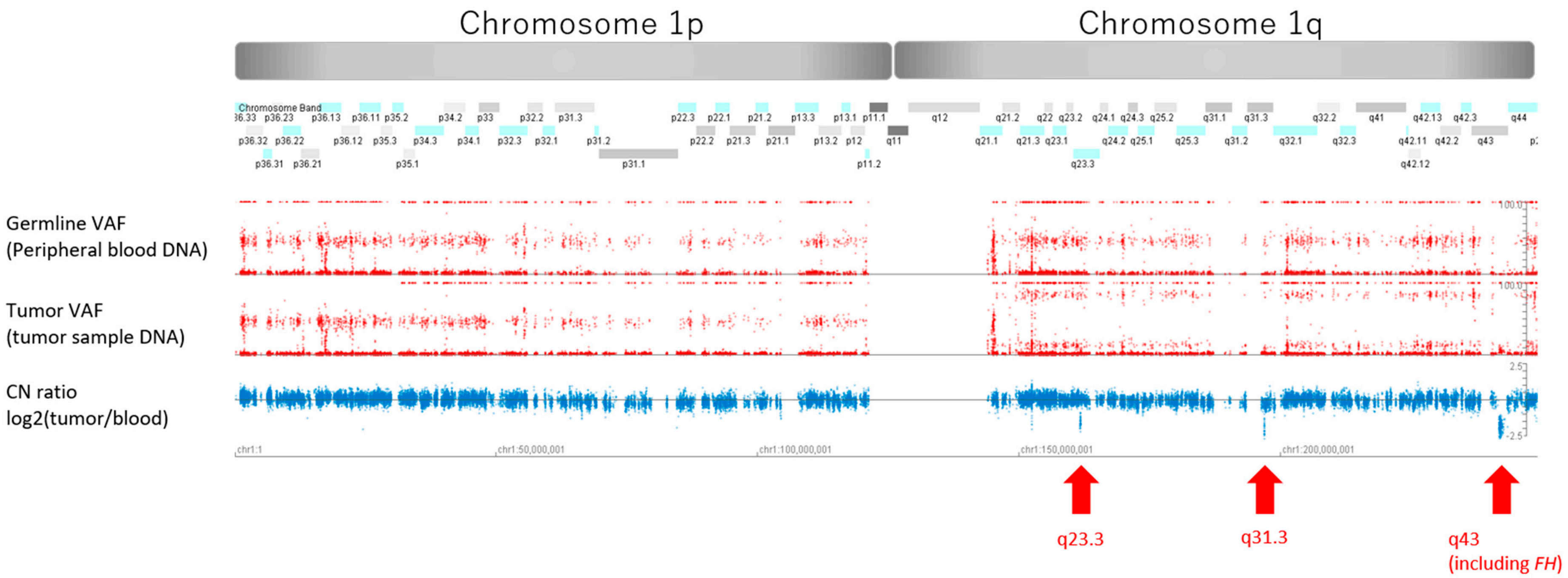

Figure S2. Results of germline and tumor profiling via exome sequencing. Shown is an overall view of chromosome 1 using a genome browser. The VAF pattern is split in a wide area of chromosome 1q in tumor tissue cells, suggesting that it is attributable to loss of heterozygosity. Furthermore, the CN ratio confirms the disappearance of the expression of the q23.1, q31.3, and q43 (including FH) regions (red arrows). CN, copy number; VAF, variant allele frequency; FH, gene encoding fumarate hydratase.
